# Supplementary material for: Single-cell sequencing of neonatal uterus reveals an Misr2+ endometrial progenitor indispensable for fertility
Source: eLife. 2019 Jun 24;8:e46349. doi: 10.7554/eLife.46349 (PMC6650247; doi:10.7554/eLife.46349)
Supplement: Transparent reporting form [file elife-46349-transrepform.docx]

***eLife’s* transparent reporting form**

We encourage authors to provide detailed information *within their submission* to facilitate the interpretation and replication of experiments. Authors can upload supporting documentation to indicate the use of appropriate reporting guidelines for health-related research (see [EQUATOR Network](http://www.equator-network.org/%20)), life science research (see the [BioSharing Information Resource](https://biosharing.org/)), or the [ARRIVE guidelines](http://www.plosbiology.org/article/info:doi/10.1371/journal.pbio.1000412) for reporting work involving animal research. Where applicable, authors should refer to any relevant reporting standards documents in this form.

If you have any questions, please consult our Journal Policies and/or contact us: [editorial@elifesciences.org](mailto:editorial@elifesciences.org).

**Sample-size estimation**

- You should state whether an appropriate sample size was computed when the study was being designed
- You should state the statistical method of sample size computation and any required assumptions
- If no explicit power analysis was used, you should describe how you decided what sample (replicate) size (number) to use

Please outline where this information can be found within the submission (e.g., sections or figure legends), or explain why this information doesn’t apply to your submission:

Explicit power analyses were not employed in the calculation of sample sizes.

Number of replicates are presented in the source data of the subsequent experiments.

Three biological replicates were used for the single cell RNA sequencing experiment. The number of cells and read per cells used in the single-cell sequencing experiment was determined empirically by the feasibility and scale of the experiment to generate a representative population of cells and informative gene expression counts.

**Replicates**

- You should report how often each experiment was performed
- You should include a definition of biological versus technical replication
- The data obtained should be provided and sufficient information should be provided to indicate the number of independent biological and/or technical replicates
- If you encountered any outliers, you should describe how these were handled
- Criteria for exclusion/inclusion of data should be clearly stated
- High-throughput sequence data should be uploaded before submission, with a private link for reviewers provided (these are available from both GEO and ArrayExpress)

Please outline where this information can be found within the submission (e.g., sections or figure legends), or explain why this information doesn’t apply to your submission:

Information about the technical replicates are explained in the Materials and Methods section of each subsequent experiment, and presented in the source data.

**Statistical reporting**

- Statistical analysis methods should be described and justified
- Raw data should be presented in figures whenever informative to do so (typically when N per group is less than 10)
- For each experiment, you should identify the statistical tests used, exact values of N, definitions of center, methods of multiple test correction, and dispersion and precision measures (e.g., mean, median, SD, SEM, confidence intervals; and, for the major substantive results, a measure of effect size (e.g., Pearson's r, Cohen's d)
- Report exact p-values wherever possible alongside the summary statistics and 95% confidence intervals. These should be reported for all key questions and not only when the p-value is less than 0.05.

Please outline where this information can be found within the submission (e.g., sections or figure legends), or explain why this information doesn’t apply to your submission:

Statistical analyses for all relevant numeric data sets are summarized in the figure legends, and listed in the corresponding figure supplemental datasets (Source data).

or Materials and Methods.

(For large datasets, or papers with a very large number of statistical tests, you may upload a single table file with tests, Ns, etc., with reference to sections in the manuscript.)

**Group allocation**

- Indicate how samples were allocated into experimental groups (in the case of clinical studies, please specify allocation to treatment method); if randomization was used, please also state if restricted randomization was applied
- Indicate if masking was used during group allocation, data collection and/or data analysis

In order to generate aged-matched controls, all the newborn female siblings (mice or/and rats) were randomly allocated to the treatment (+ or – AAV9-MIS or recombinant MIS) or control groups (AVV9-empty or saline vehicle). No masking was used during group allocation, data collection and/or data analysis.

In the single cell sequencing experiment, uteri from newborn sibling female rats were similarly allocated to AAV9-MIS or AAV9-empty vector control groups, and were pooled (N=3) at the time of collection and dissociated in one reaction.

Please outline where this information can be found within the submission (e.g., sections or figure legends), or explain why this information doesn’t apply to your submission:

**Additional data files (“source data”)**

- We encourage you to upload relevant additional data files, such as numerical data that are represented as a graph in a figure, or as a summary table
- Where provided, these should be in the most useful format, and they can be uploaded as “Source data” files linked to a main figure or table
- Include model definition files including the full list of parameters used
- Include code used for data analysis (e.g., R, MatLab)
- Avoid stating that data files are “available upon request”

Please indicate the figures or tables for which source data files have been provided:

Single cell sequencing data is uploaded to Raw data and appropriate statistical analyses for all relevant numeric data sets are presented in the corresponding Figure supplemental datasets (Source data and statistics).
